# Variation in Thermal Performance of a Widespread Pathogen, the Amphibian Chytrid Fungus *Batrachochytrium dendrobatidis*

**DOI:** 10.1371/journal.pone.0073830

**Published:** 2013-09-04

**Authors:** Lisa A. Stevenson, Ross A. Alford, Sara C. Bell, Elizabeth A. Roznik, Lee Berger, David A. Pike

**Affiliations:** 1 School of Marine and Tropical Biology, James Cook University, Townsville, Queensland, Australia; 2 School of Public Health, Tropical Medicine and Rehabilitation Sciences, James Cook University, Townsville, Queensland, Australia; Smithsonian's National Zoological Park, United States of America

## Abstract

Rates of growth and reproduction of the pathogens that cause emerging infectious diseases can be affected by local environmental conditions; these conditions can thus influence the strength and nature of disease outbreaks. An understanding of these relationships is important for understanding disease ecology and developing mitigation strategies. Widespread emergence of the fungal disease chytridiomycosis has had devastating effects on amphibian populations. The causative pathogen, *Batrachochytriumdendrobatidis* (*Bd*), is sensitive to temperature, but its thermal tolerances are not well studied. We examined the thermal responses of three Bd isolates collected across a latitudinal gradient in eastern Australia. Temperature affected all aspects of *Bd* growth and reproduction that we measured, in ways that often differed among *Bd* isolates. Aspects of growth, reproduction, and their relationships to temperature that differed among isolates included upper thermal maxima for growth (26, 27, or 28°C, depending on the isolate), relationships between zoospore production and temperature, and zoospore activity and temperature. Two isolates decreased zoospore production as temperature increased, whereas the third isolate was less fecund overall, but did not show a strong response to temperature until reaching the upper limit of its thermal tolerance. Our results show differentiation in life-history traits among isolates within Australia, suggesting that the pathogen may exhibit local adaptation. An understanding of how environmental temperatures can limit pathogens by constraining fitness will enhance our ability to assess pathogen dynamics in the field, model pathogen spread, and conduct realistic experiments on host susceptibility and disease transmission.

## Introduction

Emerging infectious diseases can have devastating effects on wildlife populations [1,2], and in some cases a single disease can drive a species to extinction before the cause is identified or understood [3]. Disease emergence can be caused by human encroachment into wildlife populations, habitat degradation, the global translocation of plants and animals, and ‘spillover’ of pathogens from human and domestic animal populations to local wildlife [2,4]. Because many pathogens are sensitive to temperature, precipitation, and humidity, disease epidemics can also be triggered by anthropogenic changes in environmental conditions [1,5]. There is growing concern that climate warming and increasingly variable weather could increase pathogen development, transmission, and host susceptibility [1,4]. Understanding how pathogens respond to their environment and hosts will aid in the development of strategies to mitigate disease in wildlife populations. In many cases, however, we do not yet understand the role of climate in disease spread, or even how temperature influences pathogen functional performance [1].

To make progress in crucial research areas, including assessing pathogen dynamics in the field, modelling pathogen spread, predicting impacts, and conducting realistic experiments on host susceptibility and transmission, we need to understand how pathogens are constrained by their environment. *Batrachochytriumdendrobatidis* (*Bd*) is a parasitic fungal pathogen that causes chytridiomycosis, which has been described as the worst emerging disease affecting vertebrate biodiversity in recorded history [6]. The spread of this pathogen is widely cited as the cause of many recent enigmatic declines in amphibian populations [5–7]. The prevalence and intensity of *Bd* infections tend to be greater during cooler months of the year, and often at high-elevation sites [8–12]. This is thought to be a function of the thermal performance of the pathogen, which has been reported to grow and reproduce best under moist conditions ranging from 17–25°C [13]. This relatively narrow temperature range is similar to environmental temperatures in many high-elevation tropical rainforests, which has led to the hypothesis that climate change (via increased cloud cover in montane rainforests) could exacerbate disease outbreaks [14].

When pathogens maximize their growth at temperatures near the upper lethal limit for reproduction, very small differences in temperature can have very large effects on their prevalence and the outcomes of infections [15,16]. The upper limit for maximal *Bd* growth (25°C) is close to its upper lethal limit (28°C [13]), suggesting that the body temperature, thermal biology, and thermal environment of amphibian hosts should strongly influence the outcome of infection [17–19]. Other temperature-dependent aspects of amphibian physiology, including the immune system [20,21], could further influence the virulence of the pathogen. An incomplete understanding of the thermal response of *Bd* has limited our understanding of these issues. For example, the only study comparing *Bd* growth across its lower and upper optimal temperature range is limited by relatively coarse resolution (i.e., 4, 10, 17, 23, 25 and 28°C [13]). Missing links for *Bd* include clarifying its upper thermal limit (i.e., exploring temperature sensitivity between 25 and 28°C), and the lower limit of its thermal optimum (commonly cited as 17°C, based on comparatively reduced growth at 10°C [13]).

*Bd* has been detected on six continents, and early reports suggested that *Bd* samples collected across wide geographic and environmental ranges were similar in morphology and growth patterns [13]; these results were reinforced by genetic similarities identified by multi-locus sequence typing [22]. More recent studies ( [23–26]) suggest that the evolutionary history of the pathogen may be much more complex: strains can differ morphologically and life-history characteristics can evolve in the laboratory while strains are under culture [26,27]. Recent results additionally suggest that different geographic regions could harbour strains that have adapted to local environmental conditions, and thus differ in their functional responses to temperature. A widespread pathogen with fixed responses to temperature could have very different impacts from a widespread pathogen that has adapted to a wide range of local environments. The latter is likely to have stronger impacts on host populations, because local adaptation could increase virulence [27]. Understanding the thermal responses of *Bd* strains isolated from locations experiencing a variety of thermal conditions will provide crucial insights into its effects on amphibians.

We studied how constant temperatures influence the growth and reproduction *in vitro* of three different *Bd* isolates collected across a latitudinal gradient to: (a) examine how a range of temperatures influences the growth and reproduction of *Bd in vitro*, and whether these patterns are consistent across isolates; (b) examine the exact nature of the upper thermal limit to *Bd* growth, which ceases abruptly between 25°C and 28°C [13]; and (c) determine whether *Bd* can reproduce after exposure to temperatures just above the limit at which little or no growth occurs.

## Materials and Methods

### Thermal regimes

We examined the effects of a range of constant-temperature environments on the growth of *Bd in vitro*. Cultures were incubated simultaneously at constant temperatures in one of ten incubators (accuracy and precision to ±0.5°C) at 13, 15, 17, 19, 21, 23, 25, 26, 27 or 28°C; these temperatures encompass the optimal range (17-25°C) identified by [13] and add resolution immediately outside that range. During a pilot study, zoospores did not develop into zoosporangia in any of the three isolates when they were maintained at 29°C; therefore we chose 28°C as our maximum temperature. Incubator temperatures were recorded every 15 minutes using Thermochron iButton temperature loggers (two per incubator; Maxim Integrated Products, California, USA; factory-calibrated and accurate to ±0.5°C). Prior to analyses, we inspected these thermal data to ensure that the incubators maintained their set temperatures during the experiment.

### Culture and maintenance of *Batrachochytrium dendrobatidis*

**Figure 1:**
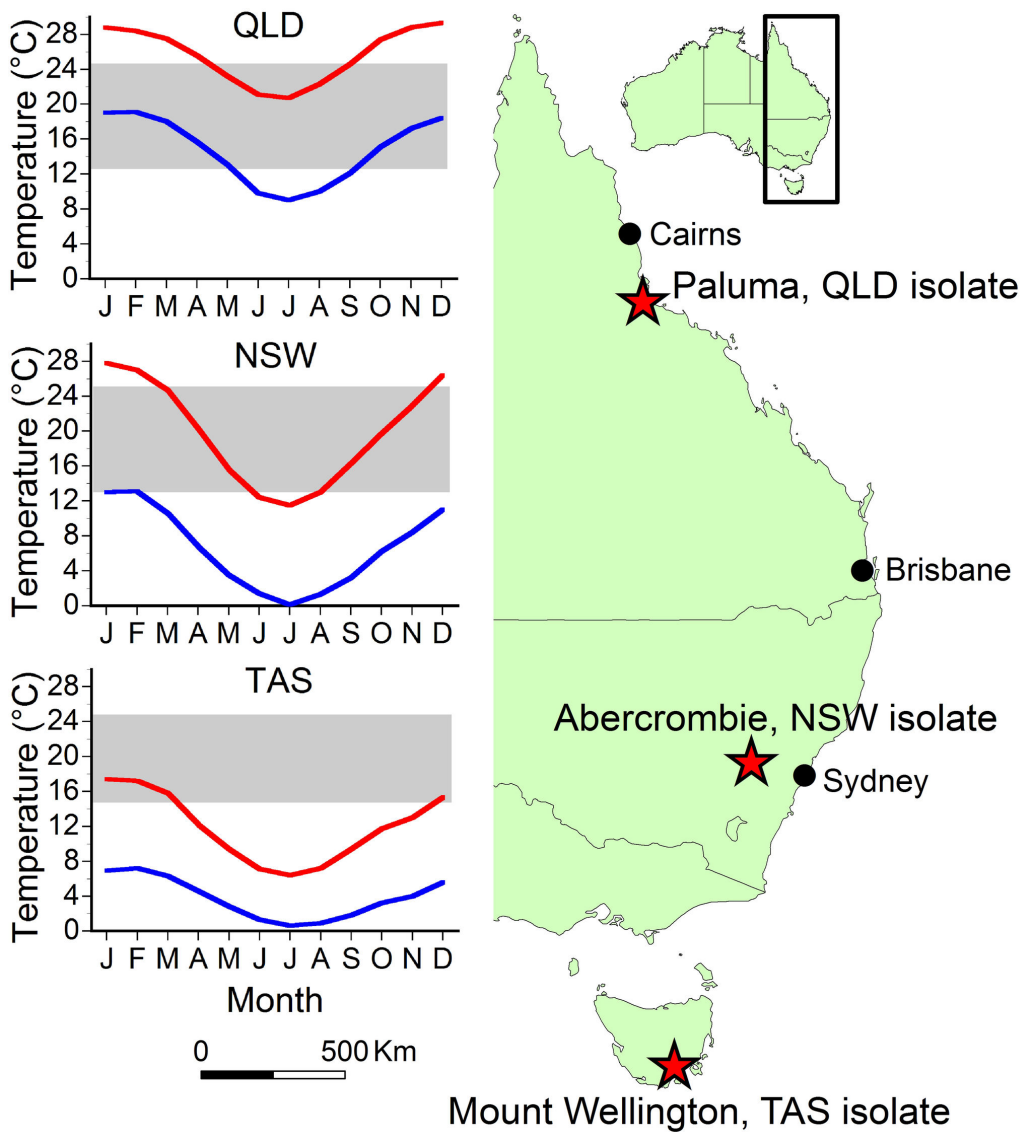
The locations of field sites from which *Bd* isolates were collected (stars), along with mean monthly maximum (red lines) and minimum (blue lines) air temperatures (from [45]). *Bd* was first reported from the Queensland site in 1990, the New South Wales site in 1997, and the Tasmania site in 2004. The temperature range tested that produced maximum growth is shaded for each isolate.

We used three Bd isolates: (1) Paluma-Lgenimaculata #2 (tadpole)-2010-CO (“QLD”), (2) AbercrombieR-Lbooroolongensis-09-LB1 (“NSW”), and (3) MtWellington-Lewingii (tadpole)-2012-RW1 (“TAS”). These isolates represent a wide latitudinal gradient spanning a range of temperatures, precipitation patterns, and pathogen histories found across eastern Australia (Figure 1). *Bd* was first documented near the QLD site in 1990, the NSW site in 1997, and the TAS site in 2004 [28]. All three were isolated (in 2009, 2010, and 2012, respectively; see below) from infected animals collected in the field which were removed to the laboratory and euthanised by bathing in 0.1% MS-222 (tricaine methanesulfonate, Sigma-Aldrich Inc., St Louis, MO 63103, USA). The tadpole isolates came from animals that had mouthpart anomalies but otherwise appeared to be healthy. Animal work was approved by the James Cook University Animal Ethics Committee (approval A1783) and conducted under permits issued by the Queensland Department of Environment and Resource Management (WITK11999612).

Whole frog skins or tadpole mouthpart sections were excised for isolation of *Bd* using the methods described by 29. The QLD isolate was obtained from a green-eyed treefrog ( *Litoria* *serrata* ) tadpole collected at Birthday Creek, near Paluma, Queensland in June 2010, and had been passaged serially *in vitro* 24 times at the time of our experiment. The NSW isolate was obtained from an adult Booroolong frog ( *L* *. booroolongensis* ) at Retreat, River near Abercrombie, New South Wales in May 2009, and had been passaged serially 16 times prior to use. The TAS isolate was obtained from a whistling treefrog ( *L* *. ewingii* ) tadpole from a pond at Mount Wellington, Tasmania in March 2012, and had been passaged eight times prior to use. Cultures were maintained in TGhL broth (8g tryptone, 1g gelatin hydrolysate, and 2g lactose in 1L distilled water) in 25cm^2^ tissue culture flasks (Techno Plastic Products, Trasadingen, Switzerland) at 4°C since isolation and passaged every three months. When in use, cultures were maintained at 21-23°C; the QLD culture was passaged weekly to maintain active growth and the NSW and TAS cultures were passaged biweekly due to slower growth. For each passage we extracted 1ml of active culture and added this to 9ml of fresh TGhL in a new tissue culture flask.

### Preparation of inocula

We inoculated TGhL agar plates (TGhL as above, with 10g bacteriological agar) with 0.75-1ml of *Bd* broth culture. Given that the isolates grew at different rates, a sufficient number of agar plates were inoculated to ensure that enough zoospores were available for the experiment (QLD: eight agar plates; NSW and TAS: 12 agar plates each). Plates were sealed with Parafilm® and incubated at 21-23°C for three (QLD) or five (NSW, TAS) days until we observed maximum zoospore production. On the same day, zoospores from all isolates were harvested by flooding the agar plates with 3ml of TGhL broth. To remove zoosporangia, the zoospore suspension was vacuum filtered through a sterile 20µm nylon filter (Spectra Mesh, Spectrum Laboratories Inc, California, USA). We determined the zoospore concentration by counting active zoospores in five 0.005mm^2^ squares in each of two counting chambers of a haemocytometer (Neubauer Improved Bright-line). Average counts were used to estimate the density of zoospores per ml of culture.

Zoospores from each isolate were re-suspended in TGhL at a concentration of 0.575x10^6^ zoospores per ml prior to inoculation into 96-well plates (Costar 3595, Corning, New York, USA). Twenty plates were each divided into seven sections: the central wells were split among the three isolates (18 replicates each), each containing 100µl *Bd*. A further three sections comprised negative controls for each isolate (six replicates each); negative controls were prepared using the same methods as used for the treatment wells, but with heat-killed *Bd* (maintained at 60°C for 45mins). The final section (24 replicates) contained 100µl TGhL broth (used only as controls to check for media contamination). The plates were replicated using three different arrangements of this design to ensure that location within the plates did not influence the growth of the isolates (Appendix S1). Two 96-well plates of different layouts were haphazardly assigned to each incubator to control for potential plate effects. In addition, we rotated the plates 180° every 24 hours within the incubators to ensure that all wells were evenly exposed to incubator temperatures.

### *Batrachochytrium dendrobatidis* growth assay

Immediately after the initial plate setup, and every 24 hours thereafter for the duration of the experiment, we measured growth of *Bd* spectrophotometrically using a Multiskan Ascent 96/384 Plate Reader (MTX Lab Systems Incorporated, Virginia, USA) at an absorbance of 492nm. Final daily absorbance values were obtained for each treatment on each day by subtracting the average optical density of the replicate negative controls from the average optical density of the replicate treatment wells. Our experiment was run for fifteen days (31 August -14 September 2012), at which time the cultures reached a stationary phase, as indicated by a plateau in optical density readings. We visually inspected the plates daily using an inverted light microscope to monitor zoospore activity and check for contamination. Contaminated wells had unusually high optical density readings accompanied by discolouration, and were excluded from analysis. To provide a visual record of growth, we photographed representative samples of each isolate from each plate on Days 8 and 14 under an inverted light microscope (using a Panasonic DMC-G1K digital camera).

### Quantifying reproductive fitness: fecundity and growth over time

We quantified the reproductive output and growth of each of our three Bd isolates over 3-4 consecutive days, starting when we observed initial zoospore release and continuing until maximum zoospore release. We measured zoospore production by sacrificing a single, randomly selected well from each isolate from each plate daily. We removed 30µl from each sacrificed well and quantified the zoospore concentration per ml (as above in *Preparation of inocula*). Prior to sacrificing these wells, each was photographed. Data from sacrificed wells were used to quantify reproductive potential only, and were not used in other analyses.

### Growth and recovery of *Bd* isolates at high temperatures

During a pilot study, we observed little growth in *Bd* cultures maintained in our highest temperature treatments (i.e., 26-28°C), and no growth in cultures maintained at 29°C. We sought to clarify the upper thermal limits of *Bd* by moving plates from our warmest treatments into a cooler environment to determine whether normal growth and development would commence. On Day 14 of our experiment, we moved the high-temperature treatments (26, 27 and 28°C) to a lower temperature (23°C), and visually monitored them for zoospore activity and growth. We continued to measure the growth of the cultures spectrophotometrically each day until the optical density readings reached a plateau (Day 34). Representative samples of each isolate from each plate were photographed on the first day of zoospore release and every day thereafter until the last day of zoospore release, and also on the day that zoospore movement ceased. We used ImageJ Version 1.44p (Rasband 2011) to measure the surface area occupied by each of 20 zoosporangia that were haphazardly chosen from the same generation within each photograph.

### Statistical analyses

Statistical analyses were performed in SYSTAT Version 13; results were considered to be significant when *P* < 0.05. We tested for differences in the growth of each *Bd* isolate among our ten constant-temperature treatments. First, we used a repeated-measures ANOVA to compare *Bd* growth rates among thermal treatments (N=10), using day as the repeated measure and optical density as the dependent variable. After confirming that the pattern of *Bd* growth over time differed among the treatments (as evidenced by a significant interaction between treatment and day), we clarified these differences by comparing the optical density values during both the logarithmic growth phase (from Day 5 of our experiment) and the stationary phase (from Day 14 of our experiment) using ANOVAs with temperature (N=10) as the factor and optical density as the dependent variable. When these ANOVAs were significant, we used Fisher’s Least Significant Difference (LSD) *post hoc* tests to clarify differences among thermal treatments. Fisher’s LSD tests maximise the power of pairwise comparisons while preserving the comparisonwise error rate at α = 0.05; the experimentwise error rate is maintained at α = 0.05 by the initial ANOVA [30].

To test for growth differences among the three isolates under our ten thermal treatments, we standardized the data for each isolate to account for any small differences in initial density estimates. We did this by subtracting from each optical density value the absolute minimum value recorded for that isolate, and dividing the remainder by the maximum value recorded for that isolate. This procedure provided a value ranging from 0-1 for each isolate for Days 5 and 14 of our experiment. We then used a two-way ANOVA with isolate and temperature as the factors and standardized optical density as the dependent variable to quantitatively test for differences in the responses of our *Bd* isolates to temperature. We used Fisher’s LSD *post hoc* tests to clarify differences among thermal treatments and isolates.

We assessed possible differences in reproductive fitness among the three Bd isolates by comparing how the isolates responded to temperature in terms of: (a) days to first zoospore production, (b) days to maximum zoospore release, (c) number of days that zoospores were active, (d) maximum number of zoospores released, and (e) sporangia size (area, µm^2^). We did this by using separate ANCOVAs to examine the possible quadratic relationship between the covariates (temperature and temperature squared) and these dependent variables, with isolate as the factor. The relationship between zoospore area and temperature was linear, and thus we used an ANCOVA with temperature as the covariate for this variable.

Initial zoospore density is inversely related to zoosporangia size, and thus small variations in starting densities among isolates could result in different sizes. Although the initial counts of active zoospores were similar among isolates, the initial ratio of dead vs live zoospores differed somewhat (QLD 0.41:1, NSW 1.1:1, and TAS 1.33:1), suggesting that a density effect would cause the TAS culture to have the smallest sporangia and QLD the largest. We used Fisher’s LSD *post hoc* tests to clarify differences among thermal treatments and isolates.

Finally, we explored the upper thermal limits of each *Bd* isolate in our thermal recovery experiment using separate repeated-measures ANOVAs for each isolate. These analyses used initial temperature as the factor (26, 27, or 28°C) and optical density on Day 14 (the last day in which these treatments were exposed to high temperatures) and Day 34 (the day we terminated the experiment) as the repeated measures. In cases of significant results, we inspected the data to determine whether optical density increased through time (indicating growth), stayed the same, or decreased (the latter two indicate that the culture had reached the stationary phase).

## Results

### Growth of *Bd* cultures over time

**Figure 2:**
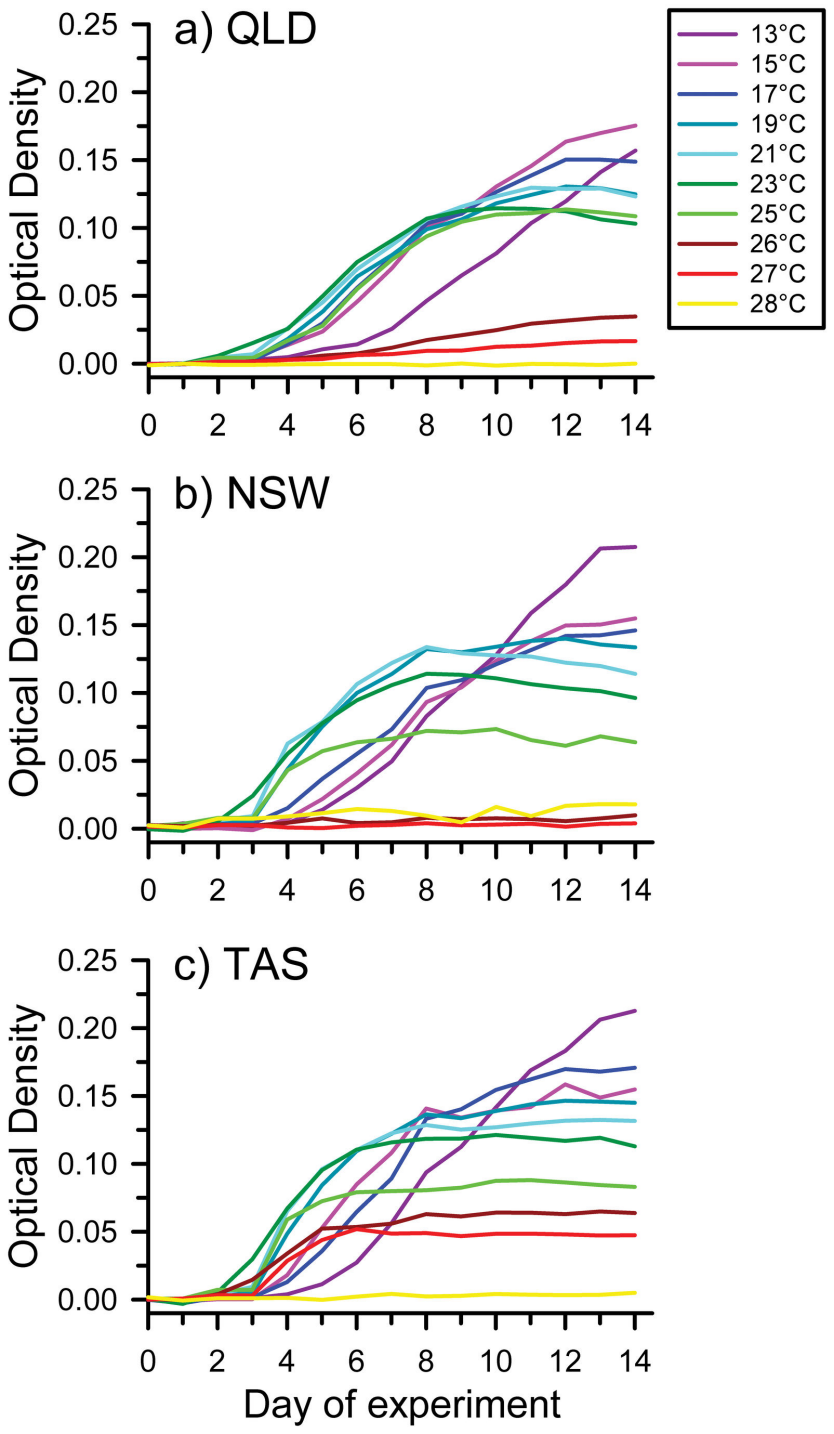
Patterns of growth of *Bd* (measured as optical density) over time (days) at constant temperatures ranging from 13–28°C, shown for isolates from (a) Queensland (QLD), (b) New South Wales (NSW), and (c) Tasmania (TAS), Australia. Shown are mean optical densities of *Bd* grown at an initial concentration of 0.575x10^6^ zoospores per ml.

**Figure 3:**
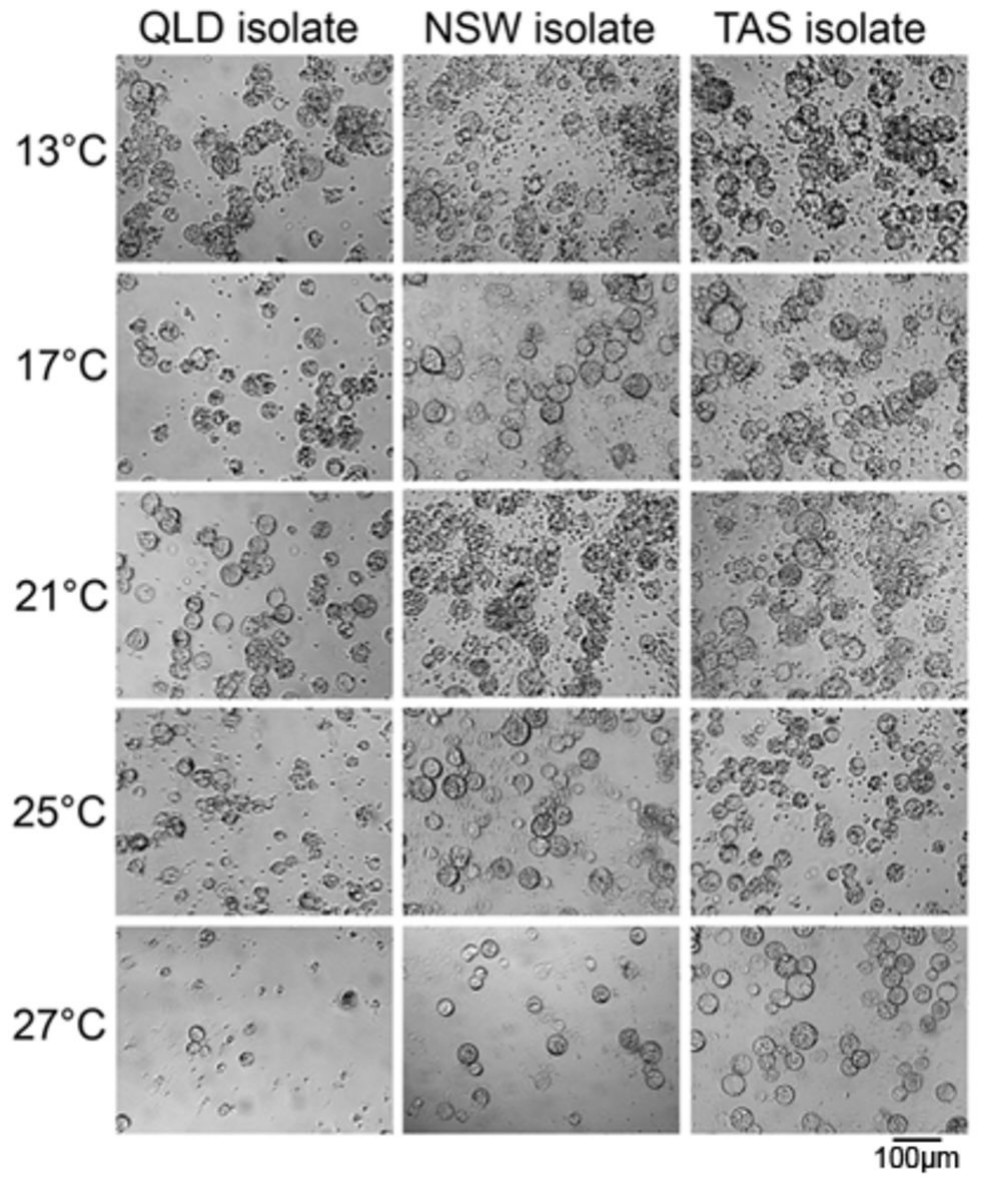
Representative photographs of three *Batrachochytrium dendrobatidis* (Bd) isolates (from Queensland, New South Wales, and Tasmania, Australia) at 13, 17, 21, 25 and 27°C. Photographs were taken on the day of maximum zoospore release.

**Figure 4:**
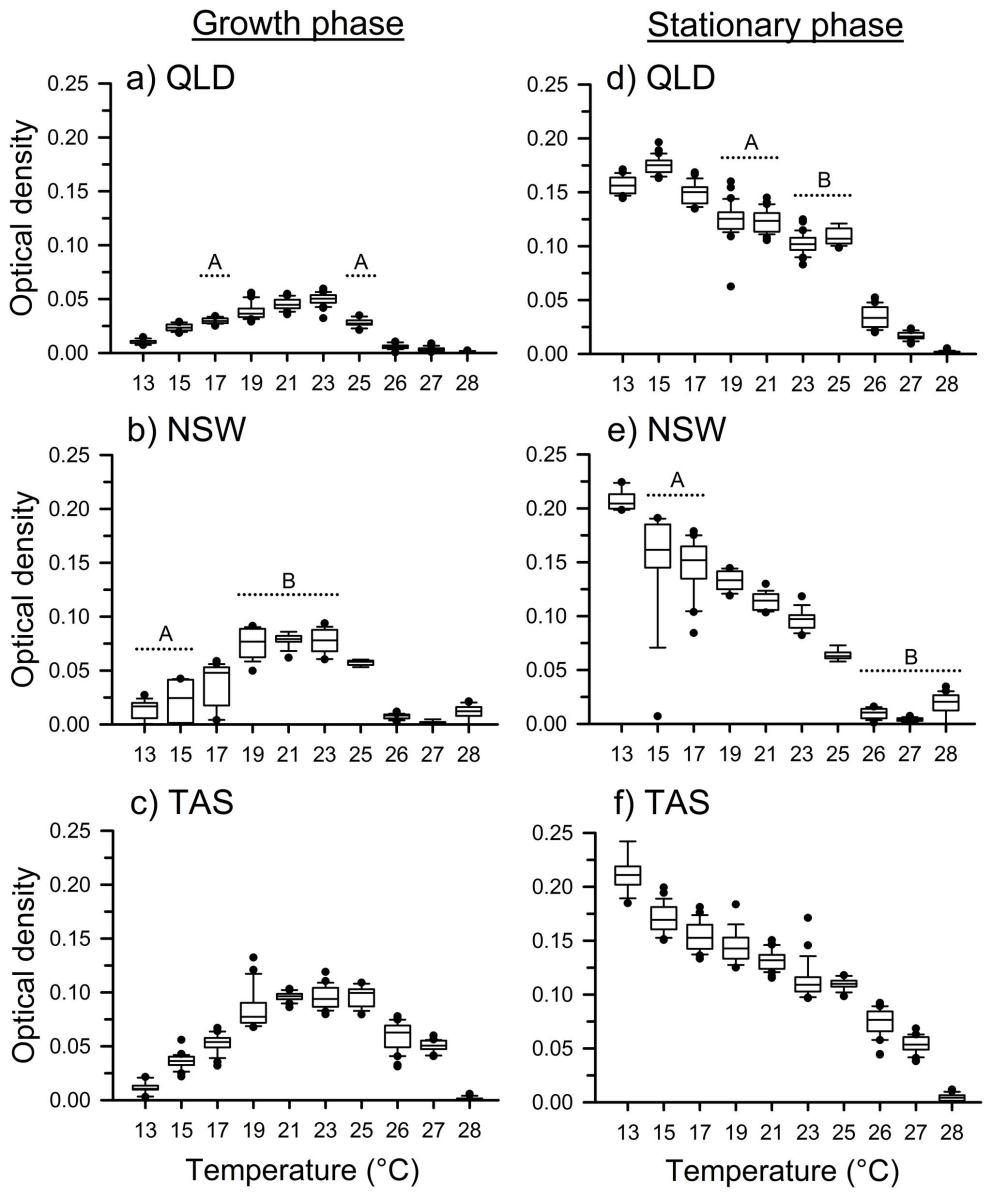
Box plots showing distributions of the optical density of *Batrachochytrium dendrobatidis* (Bd) during the logarithmic growth phase (Day 5 [Panels A-C]) and the stationary phase (Day 14 [Panels D–F]), under constant temperatures, ranging from 13–28°C, for isolates from (a, d) Queensland (QLD), (b, e) New South Wales (NSW), and (c, f) Tasmania (TAS), Australia. Shown are mean optical densities of *Bd* grown at a concentration of 0.575x10^6^ zoospores per ml. Horizontal lines indicate sets of temperatures that did not differ significantly; letters indicate groupings. Any temperature regime not included in a group differed significantly from all other temperature regimes for that isolate. See Appendix S2 for *P*-values.

Temperature and time significantly affected optical density within each of the isolates, and the effects of temperature changed through time (Table 1; Figures 2-3). Temperature significantly affected optical density during the logarithmic growth phase on Day 5 (QLD: *F*_9,273_ = 637.75, *P* < 0.0001; NSW: *F*_9,170_ = 151.21, *P* < 0.0001; TAS: *F*_9,254_ = 383.37, *P* < 0.0001; Figure 4) and when the cultures reached stationary growth on Day 14 (QLD: *F*_9,273_ = 1292.35, *P* < 0.0001; NSW: *F*_9,170_ = 314.15, *P* < 0.0001; TAS: *F*_9,254_ = 772.99, *P* < 0.0001; Figure 4). During the logarithmic growth phase of the QLD isolate, *Bd* grew most slowly (i.e., had the lowest optical density) at 26°C and 27°C, showed moderate growth at 13°C, had the highest growth between 15–25°C, and did not grow at all at 28°C (Figure 4a). By Day 14, growth in the QLD isolate differed significantly among all temperatures except 19-21°C and 23-25°C (Figure 4d). During logarithmic growth, the NSW isolate showed intermediate growth at 25°C, had the highest growth from 19°C and 23°C, slightly lower growth at other temperatures between 13–25°C, and did not grow at 26-28°C (Figure 4b). By Day 14, growth of the NSW isolate differed significantly among many of the temperatures tested, and reached its absolute maximum at 13°C (Figure 4e). During logarithmic growth, the TAS isolate showed low growth at 13°C and 15°C, moderate growth at 17°C, 26°C and 27°C, had the highest growth between 19–25°C (maximal between 21–25°C), and did not grow at all at 28°C (Figure 4c). By Day 14, growth of the TAS isolate differed significantly among many of the temperatures tested (Figure 4f); maximal optical density was reached at 13°C. Most treatments reached the stationary phase by Day 9; cool-temperature treatments (13, 15, 17 and 19°C) developed more slowly, and when the experiment was terminated on Day 14 growth was still occurring at 13°C for NSW and TAS, and at both 13°C and 15°C for QLD. See Appendix S2 for results of all pairwise comparisons among strains and temperatures.

**Table 1:** Results of repeated-measures ANOVAs on the effects of temperature on growth of *Batrachochytrium dendrobatidis* (*Bd*) in culture, shown for each isolate.

**Factor**	***F***	**df**	***P***
**QLD**			
Temperature	1431.06	9,273	<0.0001
Day	19707.05	14,3822	<0.0001
Temperature × Day	945.92	126,3822	<0.0001
**NSW**			
Temperature	194.48	9,170	<0.0001
Day	2649.45	14,2380	<0.0001
Temperature × Day	209.04	126,2380	<0.0001
**TAS**			
Temperature	514.06	9,254	<0.0001
Day	10953.66	14,3556	<0.0001
Temperature × Day	431.67	126,3556	<0.0001

### Potential effects of passage number

**Figure 5:**
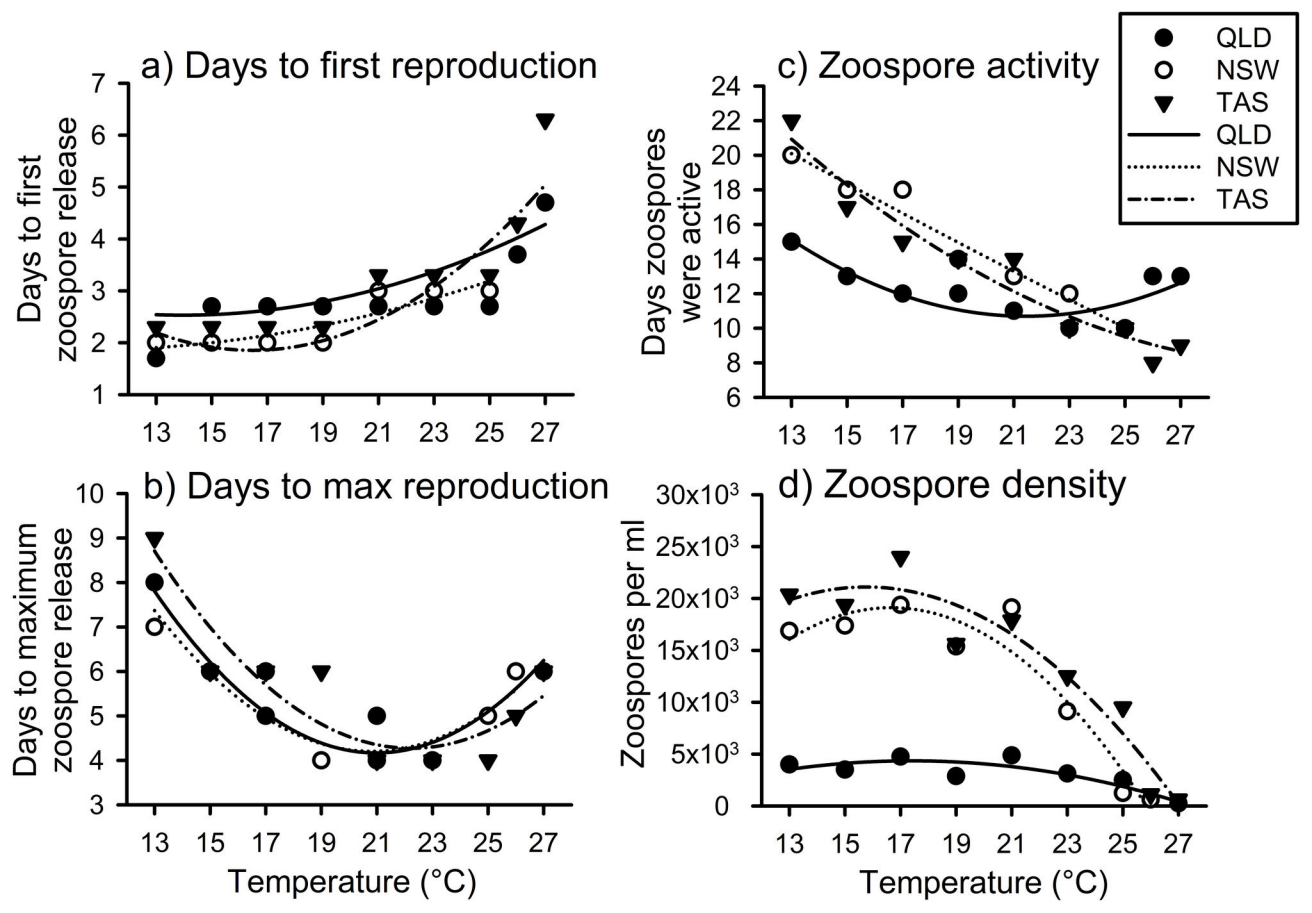
The relationships of reproductive characteristics of *Batrachochytrium dendrobatidis* (Bd), including a) days to initial zoospore production, b) days to maximum zoospore production, c) zoospore longevity, and d) maximum zoospore density. *B*. *dendrobatidis* followed similar trends across temperature treatments, but differed among the isolates. Note that in some cases the data points for different isolates are overlapping.

At 21°C and 23°C, temperatures similar to those used by [26], the differences we found among isolates were not consistent with the effects of passage number. The previous study [26] found that day of maximum zoospore release decreased and maximum zoospore density increased as passage number increased (in otherwise identical isolates). By contrast, we found that at 21°C, the number of days to maximum zoospore release were near-identical in the NSW and TAS isolates (24 and 16 passages) and slightly greater in the QLD isolate (24 passages); at 23°C all three isolates had near-identical mean days to maximum zoospore release (Figure 5b). The isolates with the fewest and medium number of passages (TAS: 8, and NSW: 16) reached much higher zoospore densities than the isolate with the greatest number (QLD: 24; Figure 5d). Neither of these results is consistent with the hypothesis that passage number affected the life history of our isolates in a manner similar to that documented by [26].

### Comparisons of thermal effects among isolates

When comparing standardised optical density values among isolates and thermal treatments, we found significant interactions during the logarithmic growth phase on Day 5 (isolate: *F*_2,697_ = 63.88, *P* < 0.0001; temperature: *F*_9,697_ = 868.31, *P* < 0.0001; isolate × temperature: *F*_18,697_ = 53.78, *P* < 0.0001) and during the stationary phase on Day 14 (isolate: *F*_2,697_ = 1956.13, *P* < 0.0001; temperature: *F*_9,697_ = 1888.16, *P* < 0.0001; isolate × temperature: *F*_18,697_= 152.99, *P* < 0.0001). During logarithmic growth, we found significant differences among strains in many of the low and high temperature comparisons (see Appendix S3 for all *P*-values). At low temperatures, there were no significant differences between optical densities of the QLD isolate at 15°C and the TAS and NSW isolates at 17°C, and between the NSW and TAS isolates at 13°C. At high temperatures, there was no significant difference between optical densities of the QLD isolate at 13°C and the NSW isolate at 28°C. Similarly, during the stationary phase, we found significant differences among strains in many of the low and high temperature comparisons (see Appendix S3 for *P*-values). At low temperatures, there were no significant differences between optical densities of the TAS isolate at 13°C and the NSW isolate at 15°C or 17°C. At high temperatures, there were no significant differences between optical densities of the QLD isolate at 27°C and the TAS isolate at 27°C, between TAS 28°C and QLD 28°C or NSW 26/27°C, or between QLD 28°C and NSW 26°C; all other strain and temperature combinations were significantly different.

### Quantifying reproductive fitness: fecundity and growth over time

The number of days to initial zoospore release did not differ significantly among the three Bd isolates (square-root transformed data; *F*_2,20_ = 1.71, *P* = 0.206), but did differ significantly among the temperature treatments (temperature: *F*_1,20_ = 3.13, *P* = 0.092; temperature squared: *F*_1,20_ = 5.99, *P* = 0.024; Figure 5a). The number of days to initial zoospore release increased from 2–3 days at the cooler temperatures to approximately five days at 27°C.

The number of days to maximum zoospore release did not differ significantly among the three Bd isolates (square-root transformed data; *F*_2,21_ = 0.22, *P* = 0.805), but did differ significantly among the temperature treatments (temperature: *F*_1,21_ = 53.03, *P* < 0.0001; temperature squared: *F*_1,20_ = 47.18, *P* < 0.0001; Figure 5b). Time to maximum zoospore release was least between 19–23°C (~4 days); time increased on both sides of this range, to 7-9 days at 13°C and six days at 26-27°C (Figure 5b).

The number of days that zoospores were active did not differ significantly among the three Bd isolates (square-root transformed data; *F*_2,20_ = 0.14, *P* = 0.155), but did differ significantly among the temperature treatments (temperature: *F*_1,21_ = 5.69, *P* = 0.027; temperature squared: *F*_1,20_ = 3.08, *P* = 0.095; Figure 5c). Zoospores were active for longest at 13°C and the period of activity decreased with rising temperature (Figure 5c).

The maximum number of zoospores produced differed significantly among both *Bd* isolates and temperature treatments (square-root transformed data; isolate: *F*_2,21_ = 19.28, *P* < 0.001; temperature: *F*_1,21_ = 11.67, *P* = 0.003; temperature squared: *F*_1,21_ = 16.93, *P* < 0.001; Figure 5d). There was no significant difference between the maximum number of zoospores produced by the NSW and TAS isolates (Fisher’s LSD: *P* = 0.235), although both produced significantly more zoospores than the QLD isolate (Fisher’s LSD: *P* < 0.001 for both comparisons). Overall, zoospore production was high at temperatures between 13–15°C, peaked at 17°C, and then declined towards zero as temperature increased towards 28°C; no zoospores were produced by the NSW isolate at 27°C (Figure 5d).

**Figure 6:**
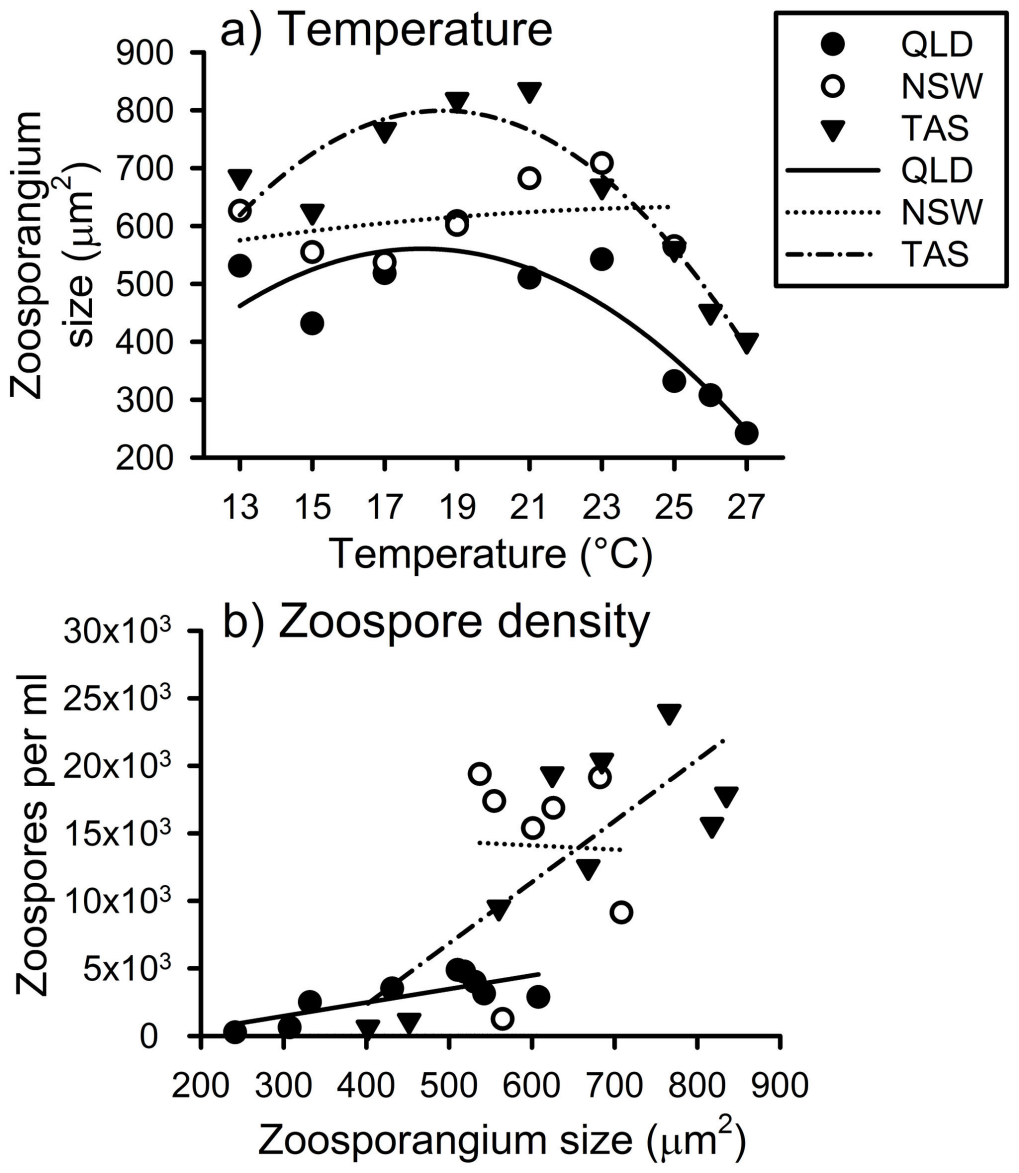
The relationships of *Batrachochytrium dendrobatidis* (*Bd*) zoosporangia sizes with a) temperature, and b) zoospore density.

Zoosporangium size (measured as area) differed significantly among both *Bd* isolates and temperature treatments (isolate: *F*_2,938_ = 47.78, *P* < 0.0001; temperature: *F*_1,938_ = 42.64, *P* < 0.0001; Figure 6a). The QLD isolate had the widest range of zoosporangium sizes (and the smallest sizes, despite having reached mature size), while the NSW isolate had a very narrow range of relatively large sizes, and the TAS isolate had the largest zoosporangia on average, with substantial size variation (Figure 6b). Treatment combinations containing larger zoosporangia also contained significantly higher densities of zoospores in the QLD (R^2^ = 0.54, *F*_1,7_ = 10.3, *P* = 0.015) and TAS isolates (R^2^ = 0.62, *F*_1,7_ = 15.94, *P* = 0.005), but not in the NSW isolate (R^2^ < 0.01, *F*_1,5_ = 0.01, *P* = 0.95).

### Growth and recovery of *Bd* isolates at high temperatures

**Figure 7:**
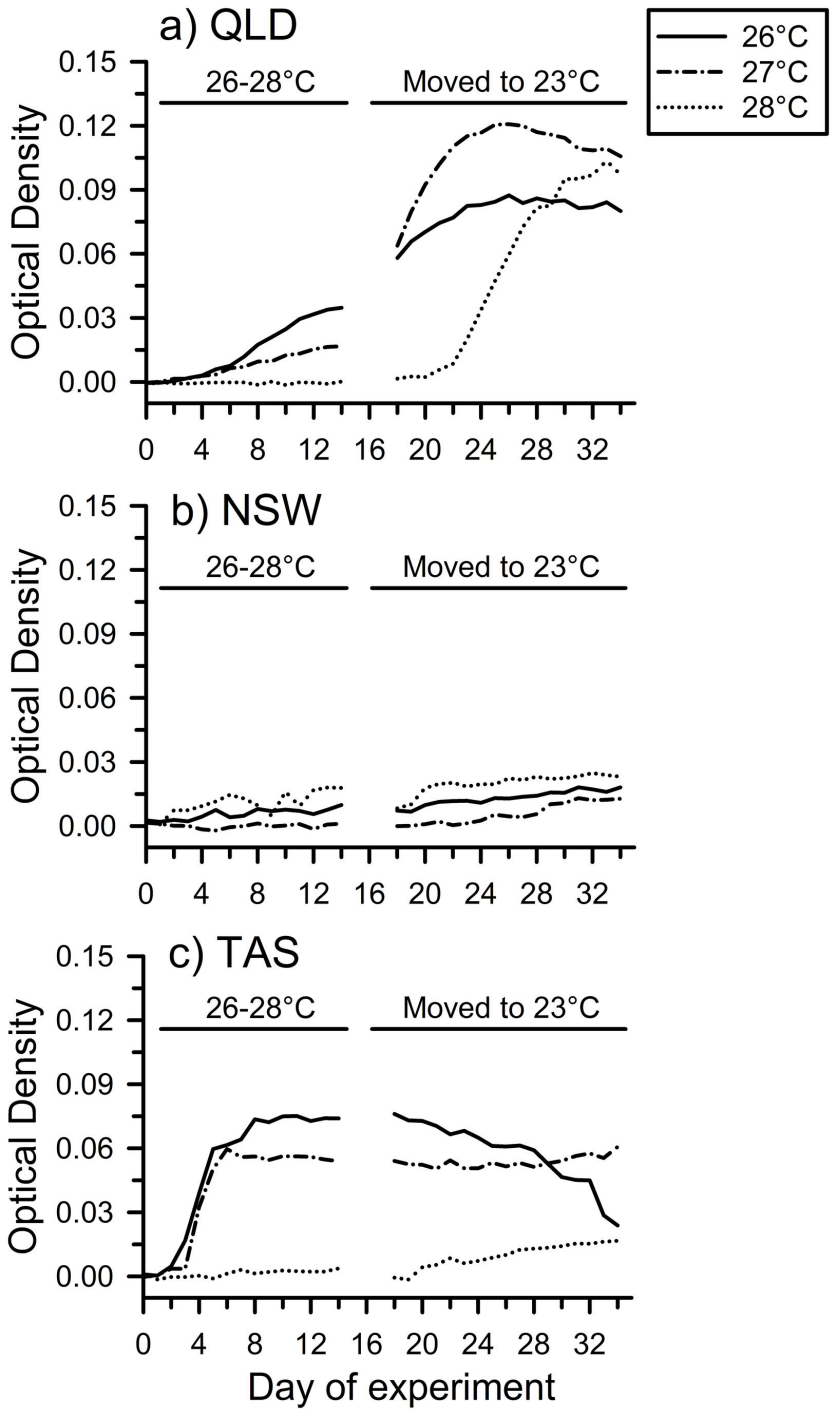
Patterns of growth of *Batrachochytrium dendrobatidis* (Bd) (measured as optical density) over time (days) at constant temperatures ranging from 26–28°C for 14 days, and maintained at 23°C thereafter, shown for isolates from (a) Queensland (QLD), (b) New South Wales (NSW), and (c) Tasmania (TAS), Australia. Shown are mean optical densities of *Bd* grown at an initial concentration of 0.575x10^6^ zoospores per ml.

Growth of the QLD isolate increased significantly over time when the high temperature treatments (26, 27, and 28°C) were moved to a lower temperature, 23°C (temperature: *F*_2,91_ = 7.23, *P* < 0.001; day: *F*_1,91_ = 630.86, *P* < 0.0001; temperature × day: *F*_2,91_ = 22.57, *P* < 0.0001); the 26°C and 27°C treatments resumed logarithmic growth within three days of being moved to 23°C, but the QLD-28°C treatment was slower to recover and resumed growth after nine days (on Day 23; Figure 7a). Although optical density of the NSW isolate increased significantly when placed in 23°C (temperature: *F*_2,68_ = 8.86, *P* = 0.71; day: *F*_1,68_ = 22.16, *P* < 0.001; temperature × day: *F*_2,68_ = 1.43, *P* = 0.25; Figure 7b), microscopic examination of cultures indicated that this was not caused by reproduction. The optical density increased slightly in all NSW temperatures due to growth of zoosporangia (Figure 7b), but zoospores were never released, so the growth of the *Bd* populations was effectively halted. Growth of the TAS isolate also differed significantly over time when the high temperature treatments were moved to 23°C (temperature: *F*_2,88_ = 60.47, *P* < 0.001; day: *F*_1,88_ = 9.80, *P* = 0.002; temperature × day: *F*_2,88_ = 40.19, *P* < 0.001; Figure 7c). At 26°C and 27°C, the first generation of zoosporangia rapidly reached maximum size and released zoospores, but those zoospores did not themselves form zoosporangia; these treatments therefore reached maximal growth before being moved to 23°C and growth did not resume at that temperature. The optical density increased slightly in TAS-28°C due to some growth of zoosporangia, but zoospores were not released at this temperature (Figure 7b). During a pilot study, no growth occurred at 29°C for any of the isolates, even when moved to 23°C after 14 days. Overall, these results suggest that the lethal maximum thermal limit is between 28–29°C for the QLD isolate, 26-27°C for the NSW isolate, and 27-28°C for the TAS isolate. For some isolates the upper temperature limit for population growth was below the lethal maximum, as both the QLD and TAS isolates did not reproduce when maintained above 25°C, and the TAS isolate did not resume reproduction after the temperature was decreased.

## Discussion

Understanding how environmental conditions influence pathogen dynamics and local adaptation is a crucial area of disease ecology research. We sought to clarify how temperature influences the growth and reproduction of three isolates of the amphibian chytrid fungus ( *Batrachochytriumdendrobatidis* ) with different histories that were collected across a wide latitudinal range, spanning most of Australia. Temperature affected growth and reproductive characteristics within all isolates, and the initial release of zoospores, zoospore longevity, and maximum zoospore production were highest in all isolates at 13-15°C. Maximum growth and reproduction were highest at cool temperatures, decreased with increasing temperature, and ceased abruptly between 26–28°C. We found a lower optimal thermal limit than earlier studies (between 13–15°C or possibly lower, depending on the isolate), and confirmed that some zoosporangium growth but minimal reproduction occurs above 25°C [13]. The lower range of the thermal optimum is supported by recent studies describing high growth and extended reproduction at temperatures below 17°C [27,31]. At low temperatures, *Bd* shows an adaptive response that improves long-term reproductive fitness as growth slows [27,31], emphasizing the need to understand reproduction across its full thermal tolerance range.

The results of our study are significant because they greatly extend, and in some cases differ from, earlier research on the basic thermal limitations of *Bd* (e.g. [13]). In contrast to earlier studies ( [13,26,32]), we found significant differences in patterns of growth among isolates that were collected from different locations and had broadly similar passage histories in the laboratory. While initial rates of growth were very similar in all treatments at 15-25°C, differences in growth patterns were obvious at the upper thermal limits for *Bd* growth: above 25°C, the NSW isolate did not grow at all, the QLD isolate grew very slowly, and the TAS isolate initially grew reasonably well but ceased growth early in our experiment because the zoospores released by the initial zoosporangia generation did not develop fully (Figure 3). The significantly greater number of zoospores produced by the NSW and TAS isolates between 13–25°C, as compared to the QLD isolate, suggests important differences in fitness. The QLD isolate produced relatively few zoospores, but was able to do so consistently across a much wider range of temperatures than the other isolates. Despite these differences, no growth was recorded at 29°C for any of the isolates, and the timing of reproduction was similar among isolates (e.g., initial and maximum zoospore release, and zoospore longevity).

Our thermal recovery experiment, in which *Bd* maintained in high temperatures (26-28°C) was transferred to 23°C, additionally suggests that the response of *Bd* to elevated temperatures is more complex than previously realised. Our QLD isolate initially maintained at 26, 27, or 28°C resumed growth and reproduction at 23°C, but significant growth was not observed after the temperature was reduced in the other two isolates. For the TAS isolate, this was because the culture had reached a stationary phase at 26°C and 27°C prior to being moved to 23°C. Pilot experiments maintaining all three of these isolates at 29°C before moving them to 23°C did not result in any growth. We conclude that the maximum thermal limit was 28-29°C for our QLD isolate, 26-27^o^C for the NSW isolate, and 27-28^o^C for the TAS isolate. These results suggest that some *Bd* isolates may be able to persist in temperatures between 25 and 28°C without reproducing or experiencing mortality, and resume growth when exposed to lower temperatures [33]. The upper lethal limit for some isolates may be as low as 26°C and for others may be at least as high as 28°C.

Our observations support earlier evidence for differences in morphology [32], reproductive output [32], and virulence [32,34–36], of *Bd* isolates collected from different geographic locations. These results also support the suggestion of [27] that *Bd* may show adaptive responses to differing thermal regimes. Such differences are supported by studies of other widespread fungal pathogens [37–40]. Differences among isolates, particularly in their thermal limitations, have important implications for amphibians, many of which maintain body temperatures within this range [16]. Even relatively small differences among species and among individuals within species in the proportion of time spent above chytrid growth thresholds can have strong effects on the probability of infection [16]; such differences are also likely to affect amphibians’ resistance to morbidity and mortality caused by chytridiomycosis [41].

Based on current limitations of our understanding of the evolutionary history and genetic diversity of *Bd*, the growth and reproductive differences among our isolates are difficult to fully explain. We present two hypotheses: (1) that differences in the laboratory history of each isolate could have impacted our experiment, and (2) that local adaptation could explain our results. The history of an isolate maintained in the laboratory may affect population growth rate [26,27], which not only influences the probability of transmission, but may also influence the severity of disease [34]. However, the differences among our isolates are not consistent with the differences we would expect based on the results of the only study that has explicitly examined the effects of passage history on *Bd* life history characteristics [26]. In fact, if similar effects of passage history were reflected in our results, the differences we observed would likely have been greater if our isolates had identical passage histories. We thus suggest that local adaptation is also a highly plausible explanation; recent genetic studies have identified high diversity among isolates collected within small geographic areas [42], as well as among strains collected from different locations on a global scale [25]. Genetic and functional (e.g. virulence) differences among *Bd* strains are supported in earlier work by [22–24,34,35]. Many pathogens are sensitive to environmental conditions; genetic and therefore functional changes can be triggered in response to temperature, and may occur even in response to temperature fluctuations over very short temporal scales [43]. It is therefore possible that each genotype of *Bd* could evolve unique temperature-dependent variations in reproduction characteristics [32]. Based on our current experimental design, we are unable to conclusively determine whether these isolates differ because of adaptation to local conditions in the wild (e.g., driven by different pathogen histories at our study sites) or due to adaptation in the laboratory (e.g., caused by different collection dates and passage histories). Disentangling these hypotheses is challenging, because it would require the simultaneous collection of a new set of isolates from a wide geographic range, maintaining these under identical laboratory conditions (including passage rates), and repeating temperature experiments; the different growth rates that we observed among individual isolates in our study would make synchronous passaging of the three cultures extremely difficult.

Why have the differences we observed among our isolates not been observed in other studies? These differences are relatively subtle, and thus earlier studies may not have been able to document patterns of growth and reproduction to the extent that we did here. In addition, the number of incubators required to simultaneously culture *Bd* at many different temperatures are not commonly available, and limit the scope of many *in vitro* experiments. In conclusion, we found that the growth and reproduction of *Bd* differs significantly over small temperature intervals at constant temperatures, and can vary among isolates collected from across a wide latitudinal gradient. Our results suggest that the upper limit for *Bd* population growth can be lower than the lethal limit (28°C) commonly inferred from the results of [13] and that the lower thermal optimum for *Bd* growth in our isolates lies at or below 13-15°C (at least 2-4°C lower than reported by [13]). Differences among isolates can be explained by either (1) differences in laboratory histories, or (2) local adaptation and genetic variation. An understanding of the environmental limitations of pathogens, and how this influences growth and reproduction, has important implications for predicting when and where disease outbreaks will occur, and for better informed field and laboratory studies (e.g., using mechanistic modelling [44]).

## Supporting Information

Appendix S1 **The three different arrangements of 96-well plates used in our experiments: Plate A, Plate B, and Plate C.** Two of these plate arrangements (A and B; A and C; or B and C) were haphazardly assigned to each thermal treatment. Each numbered column (labelled 1-12) contains eight wells arranged in rows (A–H).
(DOC)

Appendix S2 **Fisher’s LSD post-hoc results for ANOVAs comparing optical densities among temperatures, analysed separately for each isolate during the logarithmic growth phase (Day 5) and the stationary phase (Day 14).**
(DOC)

Appendix S3 **Fisher’s LSD post-hoc results for ANOVAs comparing standardised optical densities among temperatures for each isolate during the logarithmic growth phase (Day 5) and the stationary phase (Day 14).**
(DOC)
