# Supplementary figures and images for: Variation in Thermal Performance of a Widespread Pathogen, the Amphibian Chytrid Fungus Batrachochytrium dendrobatidis
Source: PLoS One. 2013 Sep 4;8(9):e73830. doi: 10.1371/journal.pone.0073830 (PMC3762749; doi:10.1371/journal.pone.0073830)

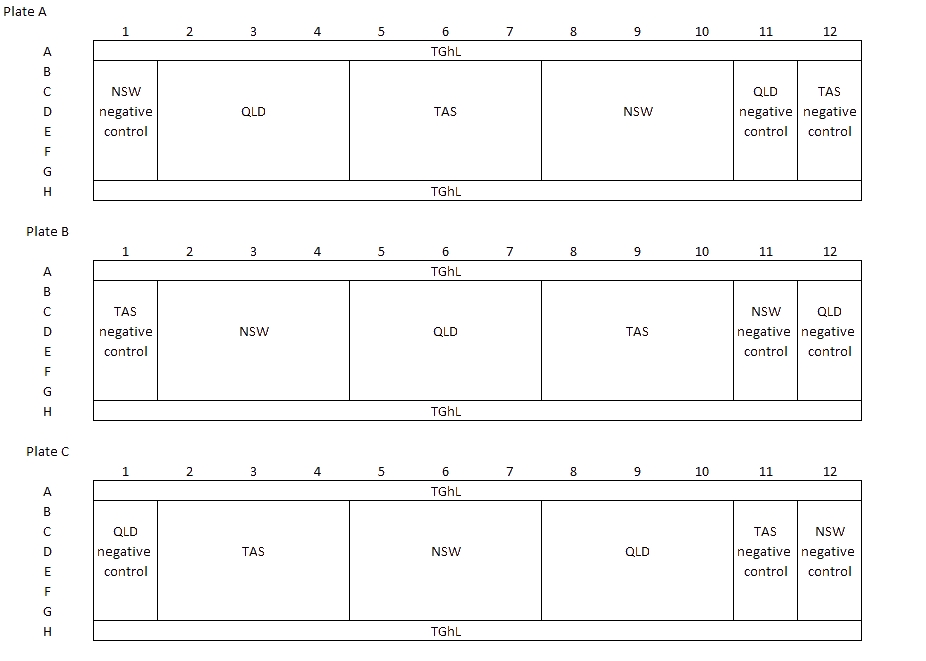

Supplement: Appendix S1 — The three different arrangements of 96-well plates used in our experiments: Plate A, Plate B, and Plate C. Two of these plate arrangements (A and B; A and C; or B and C) were haphazardly assigned to each thermal treatment. Each numbered column (labelled 1-12) contains eight wells arranged in rows (A–H). (DOC) [file pone.0073830.s001.doc]
