# Supplementary material for: Variation in Thermal Performance of a Widespread Pathogen, the Amphibian Chytrid Fungus Batrachochytrium dendrobatidis
Source: PLoS One. 2013 Sep 4;8(9):e73830. doi: 10.1371/journal.pone.0073830 (PMC3762749; doi:10.1371/journal.pone.0073830)
Supplement: Appendix S2 — Fisher’s LSD post-hoc results for ANOVAs comparing optical densities among temperatures, analysed separately for each isolate during the logarithmic growth phase (Day 5) and the stationary phase (Day 14). (DOC) [file pone.0073830.s002.doc]

Logarithmic growth phase (Day 5): QLD

|  | **15°C** | **17°C** | **19°C** | **21°C** | **23°C** | **25°C** | **26°C** | **27°C** | **28°C** |
| --- | --- | --- | --- | --- | --- | --- | --- | --- | --- |
| **13°C** | <0.001 | <0.001 | <0.001 | <0.001 | <0.001 | <0.001 | <0.001 | <0.001 | <0.001 |
| **15°C** |  | <0.001 | <0.001 | <0.001 | <0.001 | 0.001 | <0.001 | <0.001 | <0.001 |
| **17°C** |  |  | <0.001 | <0.001 | <0.001 | **0.144** | <0.001 | <0.001 | <0.001 |
| **19°C** |  |  |  | <0.001 | <0.001 | <0.001 | <0.001 | <0.001 | <0.001 |
| **21°C** |  |  |  |  | <0.001 | <0.001 | <0.001 | <0.001 | <0.001 |
| **23°C** |  |  |  |  |  | <0.001 | <0.001 | <0.001 | <0.001 |
| **25°C** |  |  |  |  |  |  | <0.001 | <0.001 | <0.001 |
| **26°C** |  |  |  |  |  |  |  | 0.015 | <0.001 |
| **27°C** |  |  |  |  |  |  |  |  | <0.001 |

Logarithmic growth phase (Day 5): NSW

|  | **15°C** | **17°C** | **19°C** | **21°C** | **23°C** | **25°C** | **26°C** | **27°C** | **28°C** |
| --- | --- | --- | --- | --- | --- | --- | --- | --- | --- |
| **13°C** | **0.058** | <0.001 | <0.001 | <0.001 | <0.001 | <0.001 | <0.001 | **0.088** | **0.505** |
| **15°C** |  | <0.001 | <0.001 | <0.001 | <0.001 | <0.001 | <0.001 | <0.001 | 0.005 |
| **17°C** |  |  | <0.001 | <0.001 | <0.001 | <0.001 | <0.001 | <0.001 | <0.001 |
| **19°C** |  |  |  | **0.377** | **0.521** | <0.001 | <0.001 | <0.001 | <0.001 |
| **21°C** |  |  |  |  | **0.803** | <0.001 | <0.001 | <0.001 | <0.001 |
| **23°C** |  |  |  |  |  | <0.001 | <0.001 | <0.001 | <0.001 |
| **25°C** |  |  |  |  |  |  | <0.001 | <0.001 | <0.001 |
| **26°C** |  |  |  |  |  |  |  | 0.029 | 0.001 |
| **27°C** |  |  |  |  |  |  |  |  | **0.225** |

Logarithmic growth phase (Day 5): TAS

|  | **15°C** | **17°C** | **19°C** | **21°C** | **23°C** | **25°C** | **26°C** | **27°C** | **28°C** |
| --- | --- | --- | --- | --- | --- | --- | --- | --- | --- |
| **13°C** | <0.001 | <0.001 | <0.001 | <0.001 | <0.001 | <0.001 | <0.001 | <0.001 | <0.001 |
| **15°C** |  | <0.001 | <0.001 | <0.001 | <0.001 | <0.001 | <0.001 | <0.001 | <0.001 |
| **17°C** |  |  | <0.001 | <0.001 | <0.001 | <0.001 | **0.335** | 0.007 | <0.001 |
| **19°C** |  |  |  | <0.001 | <0.001 | <0.001 | <0.001 | <0.001 | <0.001 |
| **21°C** |  |  |  |  | **0.761** | **0.944** | <0.001 | <0.001 | <0.001 |
| **23°C** |  |  |  |  |  | **0.858** | <0.001 | <0.001 | <0.001 |
| **25°C** |  |  |  |  |  |  | <0.001 | <0.001 | <0.001 |
| **26°C** |  |  |  |  |  |  |  | <0.001 | <0.001 |
| **27°C** |  |  |  |  |  |  |  |  | <0.001 |

Stationary phase (Day 14): QLD

|  | **15°C** | **17°C** | **19°C** | **21°C** | **23°C** | **25°C** | **26°C** | **27°C** | **28°C** |
| --- | --- | --- | --- | --- | --- | --- | --- | --- | --- |
| **13°C** | <0.001 | 0.002 | <0.001 | <0.001 | <0.001 | <0.001 | <0.001 | <0.001 | <0.001 |
| **15°C** |  | <0.001 | <0.001 | <0.001 | <0.001 | <0.001 | <0.001 | <0.001 | <0.001 |
| **17°C** |  |  | <0.001 | <0.001 | <0.001 | <0.001 | <0.001 | <0.001 | <0.001 |
| **19°C** |  |  |  | **0.501** | <0.001 | <0.001 | <0.001 | <0.001 | <0.001 |
| **21°C** |  |  |  |  | <0.001 | <0.001 | <0.001 | <0.001 | <0.001 |
| **23°C** |  |  |  |  |  | **0.066** | <0.001 | <0.001 | <0.001 |
| **25°C** |  |  |  |  |  |  | <0.001 | <0.001 | <0.001 |
| **26°C** |  |  |  |  |  |  |  | <0.001 | <0.001 |
| **27°C** |  |  |  |  |  |  |  |  | <0.001 |

Stationary phase (Day 14): NSW

|  | **15°C** | **17°C** | **19°C** | **21°C** | **23°C** | **25°C** | **26°C** | **27°C** | **28°C** |
| --- | --- | --- | --- | --- | --- | --- | --- | --- | --- |
| **13°C** | <0.001 | <0.001 | <0.001 | <0.001 | <0.001 | <0.001 | <0.001 | <0.001 | <0.001 |
| **15°C** |  | **0.136** | 0.001 | <0.001 | <0.001 | <0.001 | <0.001 | <0.001 | <0.001 |
| **17°C** |  |  | 0.029 | <0.001 | <0.001 | <0.001 | <0.001 | <0.001 | <0.001 |
| **19°C** |  |  |  | 0.001 | <0.001 | <0.001 | <0.001 | <0.001 | <0.001 |
| **21°C** |  |  |  |  | 0.002 | <0.001 | <0.001 | <0.001 | <0.001 |
| **23°C** |  |  |  |  |  | <0.001 | <0.001 | <0.001 | <0.001 |
| **25°C** |  |  |  |  |  |  | <0.001 | <0.001 | <0.001 |
| **26°C** |  |  |  |  |  |  |  | **0.229** | 0.005 |
| **27°C** |  |  |  |  |  |  |  |  | **0.098** |

Stationary phase (Day 14): TAS

|  | **15°C** | **17°C** | **19°C** | **21°C** | **23°C** | **25°C** | **26°C** | **27°C** | **28°C** |
| --- | --- | --- | --- | --- | --- | --- | --- | --- | --- |
| **13°C** | <0.001 | <0.001 | <0.001 | <0.001 | <0.001 | <0.001 | <0.001 | <0.001 | <0.001 |
| **15°C** |  | <0.001 | <0.001 | <0.001 | <0.001 | <0.001 | <0.001 | <0.001 | <0.001 |
| **17°C** |  |  | 0.003 | <0.001 | <0.001 | <0.001 | <0.001 | <0.001 | <0.001 |
| **19°C** |  |  |  | <0.001 | <0.001 | <0.001 | <0.001 | <0.001 | <0.001 |
| **21°C** |  |  |  |  | <0.001 | <0.001 | <0.001 | <0.001 | <0.001 |
| **23°C** |  |  |  |  |  | **0.466** | <0.001 | <0.001 | <0.001 |
| **25°C** |  |  |  |  |  |  | <0.001 | <0.001 | <0.001 |
| **26°C** |  |  |  |  |  |  |  | <0.001 | <0.001 |
| **27°C** |  |  |  |  |  |  |  |  | <0.001 |
