# Supplementary material for: Variation in Thermal Performance of a Widespread Pathogen, the Amphibian Chytrid Fungus Batrachochytrium dendrobatidis
Source: PLoS One. 2013 Sep 4;8(9):e73830. doi: 10.1371/journal.pone.0073830 (PMC3762749; doi:10.1371/journal.pone.0073830)
Supplement: Appendix S3 — Fisher’s LSD post-hoc results for ANOVAs comparing standardised optical densities among temperatures for each isolate during the logarithmic growth phase (Day 5) and the stationary phase (Day 14). (DOC) [file pone.0073830.s003.doc]

Logarithmic growth phase (Day 9): QLD vs NSW

|  | | **QLD** | | | | | | | | | |
| --- | --- | --- | --- | --- | --- | --- | --- | --- | --- | --- | --- |
|  |  | **13°C** | **15°C** | **17°C** | **19°C** | **21°C** | **23°C** | **25°C** | **26°C** | **27°C** | **28°C** |
| **NSW** | **13°C** | **0.91** | <0.001 | <0.001 | <0.001 | <0.001 | <0.001 | <0.001 | <0.001 | 0.006 | <0.001 |
|  | **15°C** | 0.004 | <0.001 | <0.001 | <0.001 | <0.001 | <0.001 | 0.007 | <0.001 | <0.001 | <0.001 |
|  | **17°C** | <0.001 | **0.377** | <0.001 | <0.001 | <0.001 | <0.001 | **0.074** | <0.001 | <0.001 | <0.001 |
|  | **19°C** | <0.001 | <0.001 | <0.001 | <0.001 | 0.005 | **0.859** | <0.001 | <0.001 | <0.001 | <0.001 |
|  | **21°C** | <0.001 | <0.001 | <0.001 | <0.001 | <0.001 | **0.206** | <0.001 | <0.001 | <0.001 | <0.001 |
|  | **23°C** | <0.001 | <0.001 | <0.001 | <0.001 | <0.001 | **0.385** | <0.001 | <0.001 | <0.001 | <0.001 |
|  | **25°C** | <0.001 | <0.001 | <0.001 | **0.808** | <0.001 | <0.001 | <0.001 | <0.001 | <0.001 | <0.001 |
|  | **26°C** | <0.001 | <0.001 | <0.001 | <0.001 | <0.001 | <0.001 | <0.001 | **0.238** | 0.003 | **0.107** |
|  | **27°C** | 0.003 | <0.001 | <0.001 | <0.001 | <0.001 | <0.001 | <0.001 | 0.033 | **0.763** | <0.001 |
|  | **28°C** | **0.219** | <0.001 | <0.001 | <0.001 | <0.001 | <0.001 | <0.001 | <0.001 | 0.034 | <0.001 |

Logarithmic growth phase (Day 9): QLD vs TAS

|  | | **QLD** | | | | | | | | | |
| --- | --- | --- | --- | --- | --- | --- | --- | --- | --- | --- | --- |
|  |  | **13°C** | **15°C** | **17°C** | **19°C** | **21°C** | **23°C** | **25°C** | **26°C** | **27°C** | **28°C** |
| **TAS** | **13°C** | 0.043 | <0.001 | <0.001 | <0.001 | <0.001 | <0.001 | <0.001 | 0.012 | **0.365** | <0.001 |
|  | **15°C** | <0.001 | <0.001 | <0.001 | <0.001 | <0.001 | <0.001 | 0.007 | <0.001 | <0.001 | <0.001 |
|  | **17°C** | <0.001 | **0.084** | 0.005 | <0.001 | <0.001 | <0.001 | **0.224** | <0.001 | <0.001 | <0.001 |
|  | **19°C** | <0.001 | <0.001 | <0.001 | **0.156** | <0.001 | <0.001 | <0.001 | <0.001 | <0.001 | <0.001 |
|  | **21°C** | <0.001 | <0.001 | <0.001 | <0.001 | **0.777** | <0.001 | <0.001 | <0.001 | <0.001 | <0.001 |
|  | **23°C** | <0.001 | <0.001 | <0.001 | <0.001 | **0.982** | <0.001 | <0.001 | <0.001 | <0.001 | <0.001 |
|  | **25°C** | <0.001 | <0.001 | <0.001 | <0.001 | **0.864** | 0.006 | <0.001 | <0.001 | <0.001 | <0.001 |
|  | **26°C** | <0.001 | **0.340** | <0.001 | <0.001 | <0.001 | <0.001 | 0.050 | <0.001 | <0.001 | <0.001 |
|  | **27°C** | <0.001 | <0.001 | **0.579** | <0.001 | <0.001 | <0.001 | **0.477** | <0.001 | <0.001 | <0.001 |
|  | **28°C** | <0.001 | <0.001 | <0.001 | <0.001 | <0.001 | <0.001 | <0.001 | **0.163** | <0.001 | **0.081** |

Logarithmic growth phase (Day 9): NSW vs TAS

|  | | **NSW** | | | | | | | | | |
| --- | --- | --- | --- | --- | --- | --- | --- | --- | --- | --- | --- |
|  |  | **13°C** | **15°C** | **17°C** | **19°C** | **21°C** | **23°C** | **25°C** | **26°C** | **27°C** | **28°C** |
| **TAS** | **13°C** | **0.091** | <0.001 | <0.001 | <0.001 | <0.001 | <0.001 | <0.001 | <0.001 | **0.539** | **0.352** |
|  | **15°C** | <0.001 | **0.085** | <0.001 | <0.001 | <0.001 | <0.001 | 0.007 | <0.001 | <0.001 | <0.001 |
|  | **17°C** | <0.001 | <0.001 | **0.476** | <0.001 | <0.001 | <0.001 | <0.001 | <0.001 | <0.001 | <0.001 |
|  | **19°C** | <0.001 | <0.001 | <0.001 | <0.001 | <0.001 | <0.001 | **0.229** | <0.001 | <0.001 | <0.001 |
|  | **21°C** | <0.001 | <0.001 | <0.001 | 0.010 | <0.001 | <0.001 | <0.001 | <0.001 | <0.001 | <0.001 |
|  | **23°C** | <0.001 | <0.001 | <0.001 | <0.001 | <0.001 | <0.001 | <0.001 | <0.001 | <0.001 | <0.001 |
|  | **25°C** | <0.001 | <0.001 | <0.001 | 0.023 | <0.001 | <0.001 | <0.001 | <0.001 | <0.001 | <0.001 |
|  | **26°C** | <0.001 | <0.001 | **0.976** | <0.001 | <0.001 | <0.001 | <0.001 | <0.001 | <0.001 | <0.001 |
|  | **27°C** | <0.001 | <0.001 | 0.004 | <0.001 | <0.001 | <0.001 | <0.001 | <0.001 | <0.001 | <0.001 |
|  | **28°C** | <0.001 | <0.001 | <0.001 | <0.001 | <0.001 | <0.001 | <0.001 | **0.944** | <0.001 | <0.001 |

Stationary phase (Day 14): QLD vs NSW

|  | | **QLD** | | | | | | | | | |
| --- | --- | --- | --- | --- | --- | --- | --- | --- | --- | --- | --- |
|  |  | **13°C** | **15°C** | **17°C** | **19°C** | **21°C** | **23°C** | **25°C** | **26°C** | **27°C** | **28°C** |
| **NSW** | **13°C** | <0.001 | <0.001 | <0.001 | 0.002 | <0.001 | <0.001 | <0.001 | <0.001 | <0.001 | <0.001 |
|  | **15°C** | <0.001 | <0.001 | <0.001 | <0.001 | <0.001 | **0.256** | 0.007 | <0.001 | <0.001 | <0.001 |
|  | **17°C** | <0.001 | <0.001 | <0.001 | <0.001 | <0.001 | <0.001 | <0.001 | <0.001 | <0.001 | <0.001 |
|  | **19°C** | <0.001 | <0.001 | <0.001 | <0.001 | <0.001 | <0.001 | <0.001 | <0.001 | <0.001 | <0.001 |
|  | **21°C** | <0.001 | <0.001 | <0.001 | <0.001 | <0.001 | <0.001 | <0.001 | <0.001 | <0.001 | <0.001 |
|  | **23°C** | <0.001 | <0.001 | <0.001 | <0.001 | <0.001 | <0.001 | <0.001 | <0.001 | <0.001 | <0.001 |
|  | **25°C** | <0.001 | <0.001 | <0.001 | <0.001 | <0.001 | <0.001 | <0.001 | <0.001 | 0.025 | <0.001 |
|  | **26°C** | <0.001 | <0.001 | <0.001 | <0.001 | <0.001 | <0.001 | <0.001 | <0.001 | <0.001 | **0.116** |
|  | **27°C** | <0.001 | <0.001 | <0.001 | <0.001 | <0.001 | <0.001 | <0.001 | <0.001 | <0.001 | <0.001 |
|  | **28°C** | <0.001 | <0.001 | <0.001 | <0.001 | <0.001 | <0.001 | <0.001 | <0.001 | <0.001 | <0.001 |

Stationary phase (Day 14): QLD vs TAS

|  | | **QLD** | | | | | | | | | |
| --- | --- | --- | --- | --- | --- | --- | --- | --- | --- | --- | --- |
|  |  | **13°C** | **15°C** | **17°C** | **19°C** | **21°C** | **23°C** | **25°C** | **26°C** | **27°C** | **28°C** |
| **TAS** | **13°C** | <0.001 | <0.001 | <0.001 | <0.001 | <0.001 | **0.051** | <0.001 | <0.001 | <0.001 | <0.001 |
|  | **15°C** | <0.001 | <0.001 | <0.001 | <0.001 | <0.001 | <0.001 | <0.001 | <0.001 | <0.001 | <0.001 |
|  | **17°C** | <0.001 | <0.001 | <0.001 | <0.001 | <0.001 | <0.001 | <0.001 | <0.001 | <0.001 | <0.001 |
|  | **19°C** | <0.001 | <0.001 | <0.001 | <0.001 | <0.001 | <0.001 | <0.001 | <0.001 | <0.001 | <0.001 |
|  | **21°C** | <0.001 | <0.001 | <0.001 | <0.001 | <0.001 | <0.001 | <0.001 | <0.001 | <0.001 | <0.001 |
|  | **23°C** | <0.001 | <0.001 | <0.001 | <0.001 | <0.001 | <0.001 | <0.001 | <0.001 | <0.001 | <0.001 |
|  | **25°C** | <0.001 | <0.001 | <0.001 | <0.001 | <0.001 | <0.001 | <0.001 | <0.001 | <0.001 | <0.001 |
|  | **26°C** | <0.001 | <0.001 | <0.001 | <0.001 | <0.001 | <0.001 | <0.001 | <0.001 | <0.001 | <0.001 |
|  | **27°C** | <0.001 | <0.001 | <0.001 | <0.001 | <0.001 | <0.001 | <0.001 | <0.001 | **0.729** | <0.001 |
|  | **28°C** | <0.001 | <0.001 | <0.001 | <0.001 | <0.001 | <0.001 | <0.001 | <0.001 | <0.001 | **0.065** |

Stationary phase (Day 14): NSW vs TAS

|  | | **NSW** | | | | | | | | | |
| --- | --- | --- | --- | --- | --- | --- | --- | --- | --- | --- | --- |
|  |  | **13°C** | **15°C** | **17°C** | **19°C** | **21°C** | **23°C** | **25°C** | **26°C** | **27°C** | **28°C** |
| **TAS** | **13°C** | <0.001 | **0.518** | **0.212** | <0.001 | <0.001 | <0.001 | <0.001 | <0.001 | <0.001 | <0.001 |
|  | **15°C** | <0.001 | <0.001 | <0.001 | 0.007 | **0.055** | <0.001 | <0.001 | <0.001 | <0.001 | <0.001 |
|  | **17°C** | <0.001 | <0.001 | <0.001 | <0.001 | **0.427** | <0.001 | <0.001 | <0.001 | <0.001 | <0.001 |
|  | **19°C** | <0.001 | <0.001 | <0.001 | <0.001 | 0.012 | **0.067** | <0.001 | <0.001 | <0.001 | <0.001 |
|  | **21°C** | <0.001 | <0.001 | <0.001 | <0.001 | <0.001 | **0.619** | <0.001 | <0.001 | <0.001 | <0.001 |
|  | **23°C** | <0.001 | <0.001 | <0.001 | <0.001 | <0.001 | <0.001 | <0.001 | <0.001 | <0.001 | <0.001 |
|  | **25°C** | <0.001 | <0.001 | <0.001 | <0.001 | <0.001 | <0.001 | 0.007 | <0.001 | <0.001 | <0.001 |
|  | **26°C** | <0.001 | <0.001 | <0.001 | <0.001 | <0.001 | <0.001 | <0.001 | <0.001 | <0.001 | <0.001 |
|  | **27°C** | <0.001 | <0.001 | <0.001 | <0.001 | <0.001 | <0.001 | 0.045 | <0.001 | <0.001 | <0.001 |
|  | **28°C** | <0.001 | <0.001 | <0.001 | <0.001 | <0.001 | <0.001 | <0.001 | **0.926** | **0.076** | <0.001 |
